# Ketamine for pain

**DOI:** 10.12688/f1000research.11372.1

**Published:** 2017-09-20

**Authors:** Kelly Jonkman, Albert Dahan, Tine van de Donk, Leon Aarts, Marieke Niesters, Monique van Velzen

**Affiliations:** 1 Department of Anesthesiology, Anesthesia & Pain Research Unit, Leiden University Medical Center, Leiden, Netherlands

**Keywords:** ketamine, pain, postoperative pain, analgesia

## Abstract

The efficacy of the *N*-methyl-D-aspartate receptor antagonist ketamine as an analgesic agent is still under debate, especially for indications such as chronic pain. To understand the efficacy of ketamine for relief of pain, we performed a literature search for relevant narrative and systematic reviews and meta-analyses. We retrieved 189 unique articles, of which 29 were deemed appropriate for use in this review. Ketamine treatment is most effective for relief of postoperative pain, causing reduced opioid consumption. In contrast, for most other indications (that is, acute pain in the emergency department, prevention of persistent postoperative pain, cancer pain, and chronic non-cancer pain), the efficacy of ketamine is limited. Ketamine’s lack of analgesic effect was associated with an increase in side effects, including schizotypical effects.

## Ketamine, a versatile drug

Ketamine is by far the most versatile drug available in anesthesia and possibly in all of medicine. Although this drug was designed over 50 years ago as a replacement for phencyclidine as a dissociate anesthetic, in recent years various new indications for ketamine have been discovered across multiple clinical settings, including anesthesia, pain medicine, and psychiatry ^1^. For example, in recent decades, low-dose ketamine has increasingly been used to treat moderate to severe acute and chronic pain ^2^, and in recent years ketamine has been applied as a potent and rapid-acting antidepressant in patients with therapy-resistant depression ^3^. Ketamine is a drug that acts at multiple targets; most importantly, it blocks the *N*-methyl-D-aspartate receptor (NMDAR). The excitatory glutamatergic NMDAR is ubiquitously distributed throughout the brain and spinal cord and plays an important role in the development and chronification of pain. However, ketamine’s molecular mechanism is not restricted to the NMDAR, and several studies indicate interactions with a series of receptor systems, including agonism at the opioid, AMPA, GABA, cholinergic, dopaminergic and innate repair receptors, and antagonism at HCN1, potassium, calcium, and sodium channels ^1, 4^.

The efficacy of ketamine as an analgesic is still under debate, especially for indications such as neuropathic and cancer pain ^2, 5^. The use of ketamine for pain management is widespread among clinicians caring for patients in pain, despite a paucity of randomized clinical trials showing its efficacy as well as a lack of support from medical societies and practice guidelines ^6^. Additionally, there is no consensus on dose or administration regimen. In this narrative review, we will consider ketamine’s pharmacology related to its use as an analgesic and discuss its use for the treatment of pain on the basis of the latest systematic reviews and meta-analyses. It is important to realize that, when used as an analgesic, ketamine is administered in subanesthetic rather than anesthetic doses. Subanesthetic doses are not well defined, but we regard intravenous doses up to 0.5–0.6 mg/kg and given over the course of 0.5–1 hour as subanesthetic.

## Pharmacology

There are some extensive recent reviews on the pharmacology of ketamine ^7– 9^. Here, we present data relevant to the use of ketamine for the treatment of acute and chronic pain. Ketamine has a chiral center within a cyclohexanone ring, and consequently the racemic mixture consists of R(−) and S(+) optical stereoisomers. The S(+) variant (esketamine or Ketanest S™) has a fourfold greater affinity for the NMDAR and is the more potent anesthetic agent ^7^, while there are indications from animal studies that the R(−) enantiomer has greater efficacy as an antidepressant ^8^. No data are available on the analgesic comparison of the S(+) versus R(−) enantiomers. Esketamine is available in various European countries, while in the rest of the world the racemic mixture is exclusively marketed under the name Ketalar™.

### Route of administration

Traditionally, ketamine has been administered via intravenous or intramuscular routes. However, alternative routes, such as oral, nasal, transdermal, subcutaneous, and rectal administration, have been described ^10^. Recently, the administration of preservative-free ketamine by inhalation was tested successfully. Preservative-free ketamine inhalation allows the rapid and safe administration of the drug without the need for intravenous access ^11^. Ketamine inhalation seems useful under special circumstances such as palliative, geriatric, and preclinical emergency care as well as administration at remote locations such as the battlefield.

### Metabolism

Ketamine undergoes extensive metabolism by liver cytochrome P450 enzymes. In humans, the major route of metabolism is *N-*demethylation to norketamine and subsequently cyclohexanone ring hydroxylation of norketamine to 4-, 5-, and 6-hydroxynorketamine ^9^. All metabolic products are glucuronidated and cleared via kidney and bile. During long-term intravenous ketamine infusion, similar concentrations of norketamine and its parent compound are present, and following the termination of infusion, ketamine concentrations decrease rapidly but norketamine concentrations remain elevated above ketamine concentrations for hours. Experimental human studies suggest that in the treatment of acute pain, ketamine is the driving compound with little or no analgesic contribution from its metabolite norketamine ^12^. Preliminary modeling studies even suggest some algesic effects from norketamine ^12^. This may explain the observation of hyperalgesia and allodynia after long-term ketamine treatment in patients with neuropathic pain ^13^.

### Pharmacokinetics and pharmacodynamics

Ketamine is a lipophilic compound that easily crosses the blood-brain barrier. This results in a rapid onset of action of acute pain relief with an estimated blood–effect site equilibration half-life of close to 1 minute. Consequently, for this end-point, ketamine’s effect is driven by its pharmacokinetics through actions at NMDAR and mu-opioid receptor ^12^. For other end-points such as relief of neuropathic pain and depression, the onset and offset of ketamine are much slower with persistent effects at times when plasma concentrations of ketamine and its metabolites are reduced below detection levels. For example, we calculated a ketamine half-life for pain relief in patients with complex regional pain syndrome (CRPS) of 10.9 ± 4.0 days ^14^. The slow onset/offset of ketamine in the treatment of chronic (neuropathic pain) may be explained by assuming that ketamine initiates a cascade of events, of which the effects persist beyond the treatment period. The initiating factor may be either the desensitization of the NMDAR or the activation of a receptor system that is involved in restoration of nociceptive homeostasis. We recently showed in rodents that activation of the innate repair receptor (IRR) by ketamine is a prerequisite for relief of neuropathic pain symptoms ^4^. The IRR is a receptor complex composed of the erythropoietin receptor and the beta-common-receptor (CD131). Activation of the IRR results in tissue repair and anti-inflammation ^15^. Endogenous activation is by erythropoietin (the natural anti-tumor necrosis factor cytokine), which is released upon tissue damage (for example, from ischemia, infection, and peripheral nerve damage). Of interest in this respect is the observation that in humans a selective peptide agonist of the IRR, ARA290 (Cibinetide™, Araim Pharmaceuticals, Inc.), produces effective and long-term relief of neuropathic symptoms in sarcoidosis patients with small fiber neuropathy, including the restoration of C-fibers in the cornea ^16, 17^. A final mechanism through which ketamine induces pain relief is the reinforcement of the endogenous pain modulatory system by activation of descending inhibitory pathways involved in analgesia at the spinal level ^18, 19^.

One major issue with the use of ketamine is the occurrence of side effects ^2^. Most important side effects include schizotypical effects (hallucinations, paranoia, derealization/depersonalization, and panic attacks), drug high, nausea/vomiting, and hypertension. These side effects can be present during administration but rapidly dissipate upon termination of treatment. Especially, the schizotypical effects limit patient (and doctor) compliance. However, at low-dose and/or by adding a benzodiazepine or an alpha-2-receptor agonist, the occurrence of side effects is limited and often well tolerated by the patient. Additionally, long-term use of ketamine is associated with tissue damage (for example, ** allergic hepatitis and hemorrhagic cystitis) and possibly also cognitive defects ^2, 20^.

## Ketamine for pain

To further understand the efficacy of ketamine in the treatment of pain, we performed a literature search for systematic reviews and meta-analyses on the use of ketamine for the treatment of pain in PubMed, Web of Science, Embase, and Cochrane Library (search performed March 2017; search strategies are available from the authors). We retrieved 190 unique articles, of which 29 were deemed appropriate for use in this review ( Table 1). As it is our experience that ketamine’s efficacy differs among the different pain phenotypes, we present the results of our search separated by type of pain: acute non-postoperative pain, acute postoperative pain, prevention of chronic pain following surgery, chronic non-cancer pain, and cancer pain. In case multiple systematic reviews and meta-analyses were available, we present one or two of the latest and most complete reviews.

**Table 1.** Description of studies retrieved from the literature.

	Type of study	Included trials, number	Patients, number ^a^	Favorable ketamine outcome?	Comments
	Ketamine for acute pain (emergency or pre-hospital setting)				
Lee and Lee (2016) ^21^	Meta-analysis	6	438	No	
Motov *et al.* (2016) ^22^	Literature review	8	NA	Yes	
Sin *et al.* (2015) ^23^	Literature review	4	428	No	
Jennings *et al.* (2011) ^24^	Literature review	6	340	-	Insufficient quantitative data available
Duncan and Riley (2016) ^25^	Literature review	2	158	No	
	Ketamine for acute postoperative pain				
Michelet *et al*. (2016) ^26^	Meta-analysis	11	508	No	Study in children; trial sequential analysis
Assouline *et al.* (2016) ^27^	Meta-analysis	19	1,453	Yes	Population includes children; trial sequential analysis
Wang *et al.* (2016) ^28^	Meta-analysis	36	3,502	Yes	
Mayhood and Cress (2015) ^29^	Meta-analysis	5	NA	Yes	Ketamine preoperatively given as gargle
Cho *et al.* (2014) ^30^	Meta-analysis	24	1,257	Yes	Tonsillectomy in children
Ding *et al.* (2014) ^31^	Meta-analysis	7	492	Yes	Ketamine + morphine versus morphine
Tong *et al.* (2014) ^32^	Meta-analysis	10	522	Yes	Tonsillectomy in children
Yang *et al.* (2014) ^33^	Meta-analysis	5	266	Yes	
Mathews *et al.* (2012) ^34^	Literature review	9	NA	Yes	Study includes RCTs and systematic reviews
Dahmani *et al.* (2011) ^35^	Meta-analysis	35	1,925	Yes	Children; systemic, local, and caudal ketamine studied
Laskowski *et al.* (2011) ^36^	Meta-analysis	70	4,071	Yes	
Bell *et al.* (2009) ^37^	Meta-analysis	37	2,240	Yes	
Elia and Tramèr (2005) ^38^	Meta-analysis	53	2,839	Yes	Population includes children
Subramaniam *et al.* (2004) ^39^	Meta-analysis	37	2,385	Yes	Studies in children included
	Ketamine for prevention of persistent postoperative pain				
Klatt *et al.* (2015) ^40^	Meta-analysis	10	784	No	
McNicol *et al.* (2014) ^41^	Meta-analysis	17	1,015	No	
	Ketamine for chronic non-cancer pain				
Bell (2009) ^20^	Literature review	29	579	No	Acute but no sustained pain relief
Maher *et al.* (2017) ^42^	Literature review	26	315	No	Variable effects depending on protocol
Connolly *et al.* (2015) ^43^	Literature review	45	NA	No	Ketamine in CRPS; includes reviews, RCTs, observational studies, and case reports
Noppers *et al.* (2010) ^44^	Literature review	36	776	No	Acute pain; sustained pain relief when administered for at least 10 hours
Blonk *et al.* (2010) ^45^	Literature review	20	166	No	Studies on oral ketamine
	Ketamine for cancer pain				
Jonkman *et al.* (2017) ^5^	Literature review	4	245	No	
Bredlau *et al.* (2013) ^46^	Literature review	11	483	Effect in some patients	Includes five RCTs and six uncontrolled studies
Bell *et al.* (2012) ^47^	Meta-analysis	2	30	-	Evidence is insufficient

^a^Includes treatment and control. CRPS complex regional pain syndrome; NA, not available; RCT, randomized controlled trial.

### Ketamine effect in acute pain in an emergency or pre-hospital setting

We retrieved five systematic reviews ^21– 25^ and present the results of the most recent one. Lee and Lee (2016) retrieved six randomized controlled trials (RCTs) involving 438 patients on the use of intravenous bolus ketamine versus placebo, morphine, or fentanyl in acute pain patients aged 15 years and older and treated in the emergency department ^21^. The authors defined their primary end-point as change in pain intensity 30 minutes after treatment. The comparison of ketamine versus placebo did not reach the level of significance, although the need for rescue medication was less in studies comparing ketamine with placebo and fentanyl. Importantly, ketamine use was associated with a higher risk of neurological (relative risk (RR) 2.17, 95% confidence interval (CI) 1.37–3.42, *P* <0.001) and psychological (RR 1.36, 95% CI 4.9–29.6, *P* <0.001) events, whereas opioids were associated with a higher risk for cardiorespiratory events (RR 0.22, 95% CI 0.05–1.01, *P* = 0.05).

### Ketamine for acute postoperative pain

We retrieved 14 unique reviews on ketamine or ketamine added to an opioid (for example, in a patient-controlled analgesia (PCA) device) ^26– 39^. Three analyses were on ketamine for perioperative pain management in children, two of which were restricted to tonsillectomy. We discuss three meta-analyses:

(1) In their meta-analysis, Laskowski *et al.* (2011) included RCTs on intravenous ketamine for postoperative pain relief ^36^. They extracted 70 RCTs from the literature involving 4,701 patients. The authors performed a quantitative analysis on 47 of these studies. With ketamine (given before surgical incision, during surgery, or postoperatively), a significant reduction in total postoperative opioid use was observed (standardized difference in means (SDM) 0.63, 95% CI 0.46–0.80, *P* <0.001) with an increase in time to first analgesic rescue (SDM 0.95, 95% CI 0.55–1.26, *P* <0.001), indicative of the opioid-sparing effect of ketamine. Compared with the control group, ketamine-treated patients had more neuropsychiatric side effects ( *P* = 0.018), decreased nausea and vomiting ( *P* = 0.018), but no difference in sedation.

(2) The most recent (2016) high-quality meta-analysis on the effect of ketamine added to an opioid in a PCA device, by Assouline *et al.*, included 19 RCTs from 1,349 adults and 104 children ^27^. The primary end-points were pain intensity at 24 hours, cumulative morphine consumption at 24 hours, postoperative nausea and vomiting, respiratory adverse events, and hallucinations. The authors’ analyses show that, with ketamine, pain scores were improved by about 1 cm on the 10 cm visual analogue scale (98% CI 0.4–1.8 cm, *P* <0.001; nine trials included), morphine consumption was reduced by about 13 mg (98% CI 3–22 mg, *P* = 0.002; seven trials included), and the incidence of nausea and vomiting was less with ketamine (RR 0.56, 98% CI 0.4–0.8, *P* <0.001; seven trials included). There were no differences in occurrence of respiratory events (data from nine trials) or hallucinations (data from seven trials) between patients treated with ketamine and those who were not.

(3) Dahmani *et al*. (2011) analyzed RCTs on ketamine for perioperative pain in children ^35^. The authors included 35 RCTs in their analysis. In 18 studies ketamine (or placebo) was administered intravenously (in 985 patients), in four studies ketamine was applied topically during a tonsillectomy (225 patients), and in 13 studies ketamine was given caudally as an adjuvant to opioid or local anesthetic caudal analgesia (714 patients). Following intravenous administration, ketamine reduced postoperative pain scores 2 hours after surgery (SDM −0.45, 95% CI −0.73 to −0.16) as well as postoperative analgesic medication (SDM 0.45, 95% CI 0.29–0.72). However, in the later postoperative period, 6–24 hours after surgery, none of the comparisons was significant, indicative of a short effect of ketamine. Ketamine was not associated with increased psychomimetic side effects or nausea/vomiting. Similar observations were made for topical application of ketamine during tonsillectomy. Ketamine as an adjuvant during caudal analgesia failed to affect early and later pain scores but did increase block duration (SDM 2.26, 95% CI 1.53–2.98) and reduced analgesic requirements postoperatively (SDM 0.26, 95% CI 0.10–0.66).

### Ketamine for prevention of persistent postoperative pain

We retrieved two meta-analyses on this topic. Klatt *et al.* (2015) ^40^ identified 10 RCTs (784 patients) on the effect of intra- and postoperative intravenous ketamine on chronic pain after surgery. Three RCTs (303 patients) had a positive outcome, and the remaining seven showed no effect from ketamine on persistent pain. The meta-analysis revealed a small positive effect from ketamine on pain at rest 1 month after surgery (RR 0.52, 95% CI 0.27–0.97) but not for pain in motion (RR 0.79, 95% CI 0.53–1.16). No effect from ketamine was observed at 3, 6, or 12 months after surgery on pain at rest or in motion. McNicol *et al*. (2014) ^41^ included all RCTs that administered ketamine by intravenous or epidural routes. The authors included 17 RCTs in 1,015 patients receiving ketamine and 785 placebo. The main end-pint was persistent pain at 3, 6, and 12 months. At 3 months, a non-significant RR of developing persistent pain was 0.84 with 95% CI 0.70–1.01 ( *P* = 0.06) (that is, a non-significant 16% risk reduction). Overall, 28% of patients receiving ketamine versus ** 35% receiving placebo reported pain at 3 months postoperatively. Similar results were observed at 6 and 12 months.

### Ketamine for chronic (non-cancer) pain

We retrieved five descriptive reviews on ketamine for chronic pain ^20, 42– 45^. All reviews were descriptive due to the heterogeneity between studies and poor-quality meta-analyses were not possible. In 2010, Noppers *et al*. retrieved 36 prospective RCTs on ketamine effect in chronic non-cancer pain involving 776 patients ^44^. Ketamine was used for a variety of conditions, including chronic neuropathic pain, peripheral nerve injury, complex regional pain syndrome, chronic migraine, limb ischemia, fibromyalgia, spinal cord injury, and whiplash. Most studies showed analgesic effects during ketamine administration, but just four studies showed analgesic efficacy lasting more than 48 hours. The latter four studies all employed ketamine infusions of at least 10 hours. Hence, as already concluded by Bell in 2009 ^20^, ketamine can provide short-term pain relief in chronic pain. In addition, Noppers *et al*. concluded that long-term effects would require long-term infusion paradigms. It is important to realize that since 2010 just three RCTs on ketamine effect in chronic pain were published. They do not alter the current picture on efficacy of ketamine in chronic pain. All reviews highlight the occurrence of typical ketamine-related side effects.

### Ketamine for cancer pain

We retrieved three unique systematic reviews on the use of ketamine for pain relief in patients with cancer ^5, 46, 47^. In all RCTs included in these reviews, ketamine was used as an adjuvant to opioid therapy. The latest and most complete review dates from 2017 ^5^. This review includes four RCTs in 245 patients who received oral, intravenous, or subcutaneous ketamine. Two studies on epidural or intrathecal ketamine were not included in the primary analysis. Three of the four trials involving over 90% of the study population reported a negative outcome, and the pain relief from ketamine in the fourth RCT lasted no longer than 3 hours. Additionally, all reviews reported ketamine-related side effects occurring in most patients.

## Discussion

Our appraisal of narrative and systematic reviews and meta-analyses gives a rather inconsistent picture of the efficacy of ketamine for the treatment of pain. This observation is in agreement with our clinical experience that ketamine’s efficacy depends on pain phenotype. Ketamine treatment is most effective for the relief of postoperative pain causing reduced opioid consumption, especially in the first 6 postoperative hours without serious schizotypical side effects and with less nausea and vomiting. In contrast, for most other indications (that is, acute pain in the emergency department, prevention of persistent postoperative pain, cancer pain, and chronic non-cancer pain), the efficacy of ketamine is rather limited. The conclusion of most comprehensive and recent reviews is best summarized by the absence of evidence of benefit for ketamine as a single treatment or as an adjuvant to analgesic (for example, opioid) therapy for any of these later indications. Moreover, ketamine’s lack of analgesic effect was associated with an increase in side effects, including schizotypical effects such as hallucinations.

The use of ketamine in the perioperative pain setting is well established and is in agreement with the results of the published systematic reviews on this topic, as mentioned above. Still, despite a lack of evidence for other indications, ketamine remains abundantly used clinically for treatment of acute and chronic cancer and non-cancer pain. In fact, numerous case reports, case series, and open-label studies show ketamine efficacy in the amelioration of acute and chronic pain ^5, 48, 49^. Reasons for the apparent discrepancy in efficacy in RCTs versus open-label treatments are difficult to give. Various, often interacting, factors may play a role herein ^5, 48^: (i) One important issue is the duration of treatment. We previously showed that prolonging the duration of intravenous ketamine treatment to 10–100 hours increases the probability of therapeutic success ^46^. With the exception of three RCTs that employed long-term ketamine infusion schemes for treatment of neuropathic pain (see 46 and references cited therein), most studies use short-term infusion paradigms, reducing the analgesic efficacy of ketamine for chronic pain conditions. (ii) Another issue is dose. Often ketamine exposure is limited because of dose reduction in fear of side effects. This will cause analgesic responses no greater than those of placebo in the treatment of acute and chronic pain. (iii) RCTs are frequently rigidly designed in terms of ketamine titration. In real life, ketamine infusion rates are frequently changed up or down according to the level of pain and occurrence of side effects. Personalized, careful, and patient titration of the ketamine infusion rate and use of adjuvant analgesic medication will result in a comfortable pain state without menacing side effects. (iv) Ketamine has potent antidepressant properties ^3^. Since chronic pain and depression often coexist, ketamine may improve mood-related issues. Possibly by improvement of mood, pain perception may be altered in a positive way. Measurement of pain intensity by using a numeric or visual scale will not capture these important improvements in quality of life. (v) Scoring pain by using numerical or visual scales that require defining a lower (no pain) and upper (most intense pain imaginable) boundary is a complex process. It requires the activation of specific mental tasks that may be difficult in patients with chronic pain or patients who are cognitively impaired ^50, 51^. Ketamine may further impair the ability to score pain accurately because of its central effects. (vi) There is now evidence that the magnitude of the placebo effect in trials on analgesic treatment has increased over time ^52^. Consequently, larger samples are required before a positive (often small) contrast is detected between active and placebo treatment. Given the often-small ketamine RCTs, most of these studies are likely underpowered. (vii) It may well be that owing to the strict selection criteria, RCTs studying pain treatment are not representative for the real-life clinical setting. For example, patients represented in case series may have more extensive disease progression with more severe pain symptoms than patients in RCTs. (viii) Finally, publication bias may have resulted in the appearance of many positive open-label studies in the literature, possibly unintentionally balancing against the many negative RCTs.

## Conclusions

We show that ketamine efficacy for the treatment of pain is restricted to the perioperative setting. In other settings such as acute (non-surgical) pain, chronic cancer, and non-cancer pain, the evidence that ketamine is effective is lacking. Additionally, there is no evidence for a preemptive effect of ketamine on persistent postoperative pain. Reasons for the differential effect of ketamine in various types of pain are currently unknown. Possibly, in the perioperative setting, the use of ketamine in combination with or following the administration of piperidine opioids and inhalational or intravenous anesthetics provides added value that is not observed when other (non-centrally acting) analgesics are co-administered or when ketamine is administered as a mono-analgesic. Finally, a meta-analysis on ketamine efficacy was available for all indications described except chronic non-cancer pain. We argue that a high-quality meta-analysis and trial sequential analysis are needed for this specific indication, although we realize that the number of available high-quality RCTs will be limited.
